# Investigating the impact of quarantine on mental health: insights from the COVID-19 international border surveillance study in Canada

**DOI:** 10.1192/bjo.2021.977

**Published:** 2021-08-05

**Authors:** Cheryl Regehr, Vivek Goel, Eric De Prophetis, Munaza Jamil, Dominik Mertz, Laura C. Rosella, David Bulir, Marek Smieja

**Affiliations:** Factor-Inwentash Faculty of Social Work, University of Toronto, Canada; Dalla Lana School of Public Health, University of Toronto, Canada; Dalla Lana School of Public Health, University of Toronto, Canada; McMaster HealthLabs, Canada; Division of Infectious Diseases, Department of Medicine, McMaster University, Canada; Dalla Lana School of Public Health, University of Toronto, Canada; Dalla Lana School of Public Health, University of Toronto, Canada; McMaster HealthLabs, Canada; and Research Institute of St. Joe's Hamilton, Canada; McMaster HealthLabs, Canada; Division of Infectious Diseases, Department of Medicine, McMaster University, Canada; Research Institute of St. Joe's Hamilton, Canada; and Department of Pathology and Molecular Medicine, McMaster University, Canada

**Keywords:** Quarantine, mental health, COVID-19, cohort study, international travel

## Abstract

**Background:**

Nations throughout the world are imposing mandatory quarantine on those entering the country. Although such measures may be effective in reducing the importation of COVID-19, the mental health implications remain unclear.

**Aims:**

This study sought to assess mental well-being and factors associated with changes in mental health in individuals subject to mandatory quarantine following travel.

**Method:**

Travellers arriving at a large, urban international airport completed online questionnaires on arrival and days 7 and 14 of mandated quarantine. Questionnaire items, such as travel history, mental health, attitudes toward COVID-19, and protection behaviours, were drawn from the World Health Organization Survey Tool for COVID-19.

**Results:**

There was a clinically significant decline in mental health over the course of quarantine among the 10 965 eligible participants. Poor mental health was reported by 5.1% of participants on arrival and 26% on day 7 of quarantine. Factors associated with a greater decline in mental health were younger age, female gender, negative views toward quarantine measures and engaging in fewer COVID-19 prevention behaviours. For instance, travellers who stated that they rarely wore masks had nearly three times higher odds of developing poor mental health.

**Conclusions:**

Although the widespread use of quarantine may be effective in limiting the spread of COVID-19, the mental health implications are profound and have largely been ignored in policy decisions. Psychiatry has a role to play in contributing to the public policy debate to ensure that all aspects of health and well-being are reflected in decisions to isolate people from others.

As the scientific community has worked diligently to develop and produce vaccines, governments and public health agencies have been forced to rely on traditional public health approaches to limit the spread of COVID-19. These approaches have included hand-washing, mask-wearing and social distancing measures for the general population. In situations of heightened risk, community lockdowns and mandatory quarantine for individuals at greatest risk of infecting others have also been imposed. Quarantine of those directly exposed to highly contagious illnesses has been used on several occasions in recent decades; for instance, during the 2003 severe acute respiratory syndrome (SARS) outbreak in Canada^1^ and Ebola outbreaks in Africa.^2^ Unprecedented in recent history, however, has been the widespread use of quarantine for those crossing international borders. Although quarantine when strictly enforced may indeed limit the spread of disease by those known to be or suspected of being infected, the mental health consequences of its use as a broad-based strategy for travellers entering the country requires further consideration.

## Mental Health and COVID

The overall mental health consequences of COVID-19 have been profound. Cross-sectional survey studies suggest that members of the general population, and in particular young adults and women, have experienced heightened anxiety and depression during the COVID-19 pandemic.^3–7^ Studies of populations subjected to forced isolation or quarantine during COVID-19 report heightened depression, anxiety and post-traumatic stress when compared with others in the population;^8–11^ increased alcohol consumption;^12,13^ and exacerbation of physical health conditions.^14^ Longer-term outcomes of quarantine include a range of avoidance behaviours with respect to social contact, avoiding enclosed or public spaces, fear of returning to work and excessive concerns with hygiene and hand-washing.^15^ A smaller number of studies have measured mental health status at the onset and during the course of community-wide lockdowns, reporting increased distress over time.^16,17^ To date, few studies have considered the mental health implications of quarantine when mandated because of international travel.

Factors associated with higher levels of distress among those who have been quarantined for other reasons (such as exposed healthcare professionals) include fears of infection and stigma, anger and boredom, frustration with inadequate information, financial loss^9,18^ and length of quarantine.^9,10^ Some researchers have focused on beliefs and attitudes as factors associated with mental health distress among those in quarantine. For instance, survey research in Italy has found that those in regions with lower COVID-19 contagion rates reported higher levels of quarantine distress than those in high contagion areas, a finding that the researchers attributed to a sense of justice or proportionality.^10^ Similarly, positive attitudes toward quarantine measures and trust in institutions have been related to lower levels of mental health distress among those in quarantine.^11^ These same attitudes affect quarantine compliance.^1,19^

The aim of this study was to assess the change in mental health status in individuals subject to mandatory quarantine following travel, and to determine factors associated with changes in mental health.

## Method

This study is based on a prospective cohort study of arriving international travellers at terminal 1 of Pearson International Airport in Toronto, Canada, between 3 September 2020 and 31 October 2020.^20^ At the time of the study, all arriving international passengers (with the exception of those designated essential workers) were subject to a mandatory 14-day quarantine. The quarantine could be carried out in a private residence or rented facility; individuals subject to quarantine were asked to not leave their location for any reason except for an emergency.

### Study design

The authors assert that all procedures contributing to this work comply with the ethical standards of the relevant national and institutional committees on human experimentation and with the Helsinki Declaration of 1975, as revised in 2008. All procedures involving human participants were approved by the Advara Research Ethics Board on 2 September 2020 (approval number PRO00046282). Participants provided electronic acknowledgement of informed consent.

Full details on the development of the cohort have been previously described.^20^ In brief, travellers arriving on international flights that terminated at terminal 1 of Toronto Pearson International Airport were invited to participate in a study aimed to systematically estimate the COVID-19 positivity rate of air travellers coming to Toronto, Canada, at arrival, day 7 and day 14. A further objective, reported in this paper, was to examine the impact of quarantine on the mental health of travellers. Inclusion criteria were those aged ≥18 years who had a final destination within 100 km of Toronto Pearson airport, provided consent and could speak English or French. The study's exclusion criteria were those passengers taking a connecting flight through Pearson Airport, without internet access, who exhibited symptoms of COVID-19 on arrival or who were exempted from quarantine (e.g. essential workers).

Travellers included in the study were asked to complete online questionnaires at three time points: on arrival at the airport, and day 7 and day 14 from their place of quarantine. Questionnaire items, such as travel history, mental health, attitudes toward COVID-19 protection measures, and protection behaviours, were drawn from the World Health Organization Survey Tool and Guidance for Rapid, Simple, Flexible Behavioural Insights on COVID-19.^21^

### Variables

#### Mental health

Mental health was measured with the five-item World Health Organization Well-Being Index (WHO-5), a validated tool derived from the longer ten-item version.^22^ Compared with its longer counterpart, the WHO-5 scale contains only the positively phrased items: (1) ‘I have felt cheerful and in good spirits’, (2) ‘I have felt calm and relaxed’, (3) ‘I have felt active and vigorous’, (4) ‘I woke up feeling fresh and rested’ and (5) ‘My daily life has been filled with things that interest me’. Each item is then scored from 5 (all of the time) to 0 (never), with a maximum theoretical score of 25. Individuals who score <50% (or 12 out of 25) have scores that are consistent with individuals who are considered to have poor mental health well-being.^23,24^ For this study, our primary outcome was newly developed poor mental health, which is defined as a recorded WHO-5 score of ≤12 for the first time on day 7 or 14 of quarantine. The WHO-5 is one of the most widely used screening tools for assessing subjective psychological well-being, with a sensitivity of 0.86 and a specificity of 0.81 according to a meta-analytic review.^24^

#### Attitudes and behaviours

Several attitudinal and behavioural questions were asked during quarantine to understand the prevention behaviours travellers engaged in during the pandemic, in addition to their attitudes toward COVID-19 and the mandatory quarantine. Behavioural questions were asked during day 7 of quarantine and addressed the frequency of mask-wearing, restaurant avoidance, hand-washing and visiting friends and family.

Attitudinal questions addressed quarantine difficulty, the necessity of quarantine and quarantine length. Participants were also asked to rate their anxiety about COVID-19. All of these questions were asked on both days 7 and 14 of quarantine; however, only one response was used in the analysis presented in this paper. We used the last response for the quarantine-related questions and the first response for the question on anxiety. We believe this approach to be the most reflective of the quarantine experience. Therefore, any analyses that use these variables should be viewed as cross-sectional.

### Statistical analyses

Baseline descriptive statistics and measures of independence were calculated according to available demographic and travel-related information. This study's primary outcome was newly developed poor mental health reported on days 7 and 14 of quarantine (that is, those who received a score of ≤12). Those who had poor mental health at baseline were not flagged as having newly developed poor mental health even if their status continued throughout the study.

Logistic regression models were used to examine the differences observed among those who developed poor mental health compared with those who did not. Models that adjusted for age, gender, continent of origin, beliefs about COVID-19, and infection prevention behaviours were run. The minimally adjusted model controlled for age, gender, and the continent of origin (determined through the country of origin). Unadjusted and adjusted odds ratios regarding attitudinal and behavioural variables were run separately to quantify their independent effects on the minimally adjusted model, to address the potential for collinearity.

### Imputation

As some participants did not complete all items on the questionnaires, we used multiple imputation to impute missing values. Multiple imputation is achieved by using logistic and multinomial logistic regression to create multiple data-sets of predicted values and take the average across data-sets as the final imputed value. In the case of missing country-of-origin data, a grouped imputation approach was used. Given the large variance of responses, multiple imputations were not possible. Therefore, groups of 20 travellers that arrived at the study booth at the same time were made around missing values, and the most frequent country of origin for these groups were imputed. This approach assumes that registrants usually arrive in groups because they are recruited on their respective flights.

## Results

### Study population

The baseline demographics of our cohort are displayed in Table 1. Of the 16 361 individuals who registered for the study, 10 965 individuals responded to all WHO-5 questions at least twice, to determine change in mental health well-being status throughout the study period. Study participants arrived from all continents (except Antarctica), and represented all risk categories and age groups. The highest proportion of participants were those arriving from the Americas (54%), and younger and middle age groups (74% between the ages of 18 and 49 years).

**Table 1:** Baseline characteristics by poor mental health

		New poor mental health^a^		
	Overall, *N* = 10 965	Yes, *n* = 3446^a^	No, *n* = 7519^b^	*P*-value^c^
Gender				<0.001
Female	5370	1862 (35%)	3508 (65%)	
Male	5578	1579 (28%)	3999 (72%)	
Other	17	5 (29%)	12 (71%)	
Age, years				<0.001
18–29	3451	1156 (33%)	2295 (67%)	
30–49	4711	1589 (34%)	3122 (66%)	
50–69	2617	656 (25%)	1961 (75%)	
≥70	186	45 (24%)	141 (76%)	
Continent				<0.001
Africa	450	160 (36%)	290 (64%)	
America	5928	1891 (32%)	4037 (68%)	
Asia	1465	336 (23%)	1129 (77%)	
Europe	3088	1044 (34%)	2044 (66%)	
Oceania	34	15 (44%)	19 (56%)	

a. New poor mental health is defined as a score of ≤ 12 on the five-item World Health Organization Well-Being Index, recorded for the first time on day 7 or 14 of quarantine.

b. Statistics presented as *n* (row %)

c. Statistical tests performed: *χ*^2^-test of independence

**Fig. 1:**
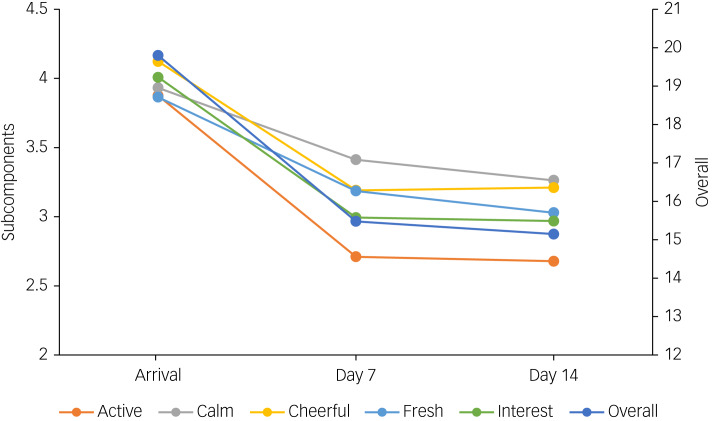
Average five-item World Health Organization Well-Being Index scores by day of quarantine.

Average WHO-5 scores by time point (arrival, day 7 and day 14) were 19.79 (95% CI 19.76−19.82), 15.48 (95% CI 15.44−15.51) and 15.15 (95% CI 15.11−15.19), respectively (see Table 2 and Fig. 1). Mean differences were statistically significant (ANOVA F-value = 2378; *P* < 0.0001) and are considered clinically meaningful.^25^ At the time of arrival, 5.1% of participants had poor mental health, as indicated by a WHO-5 score of ≤12;^23,24^ this rose to 26.0% at 7 days of quarantine and 27.0% at 14 days of quarantine. Those who developed poor mental health well-being during the period of quarantine were more likely to be in the younger and middle age groups. Furthermore, the lowest proportions of newly developed poor mental health were observed in travellers arriving from Asia (Table 1).

**Table 2:** Average five-item World Health Organization Well-Being Index scores (maximum 25) and 95% confidence intervals by time point

	Time		
	Arrival	Day 7	Day 14
Calm	3.93 (3.92–3.94)	3.41 (3.40–3.42)	3.26 (3.25–3.27)
Interest	4.01 (4.00–4.01)	2.99 (2.98–3.00)	2.97 (2.96–2.98)
Cheerful	4.12 (4.12–4.13)	3.19 (3.18–3.20)	3.21 (3.20–3.22)
Fresh	3.86 (3.86–3.87)	3.19 (3.18–3.20)	3.03 (3.02–3.04)
Active	3.88 (3.87–3.88)	2.71 (2.70–2.72)	2.68 (2.67–2.69)
Overall	19.79 (19.76–19.82)	15.48 (15.44–15.51)	15.15 (15.11–15.19)

### Logistic regression models

In the minimally adjusted model, those aged 18–29 years and 30–49 years were at 53% and 57% greater odds of developing poor well-being, respectively, during quarantine than those aged 50–69 years. Furthermore, out of all possible continents of travel, travellers arriving from Asia had reduced odds of developing reported poor well-being compared with travellers from the Americas (odds ratio 0.64, 95% CI 0.56–0.73) (Table 3).

**Table 3:** Multivariable adjusted odds ratios and 95% confidence intervals from logistic regression models

	Model 1^a^			Model 2^b^		
	Odds ratio	95% CI	*P*-value	Odds ratio	95% CI	*P*-value
Gender						
Female	−1.0	–		1.0	–	
Male	0.75	0.69–0.81	<0.001	0.77	0.71–0.83	<0.001
Other	0.73	0.23–1.98	0.6	0.70	0.22–1.90	0.5
Age, years						
50–69	1.0	–		1.0	–	
18–29	1.47	1.31–1.65	<0.001	1.53	1.36–1.71	<0.001
30–49	1.53	1.37–1.70	<0.001	1.57	1.41–1.75	<0.001
≥70	0.97	0.68–1.36	0.9	0.96	0.67–1.36	0.8
Continent						
America				1.0	–	
Asia				0.64	0.56–0.73	<0.001
Europe				1.13	1.03–1.24	0.011
Africa				1.22	0.99–1.49	0.055
Oceania				1.54	0.77–3.04	0.2

a. Model 1 controlled for age and gender.

b. Model 2 controlled for age, gender and continent of origin.

#### Attitudes about COVID-19 and control measures

Cross-sectional analyses showed that individuals with higher levels of fear regarding COVID-19 were reported by those who developed poor mental health (Table 4). Respondents who reported having anxiety about COVID-19 ‘all of the time’ had 38% higher odds of developing poor mental health well-being (adjusted odds ratio 1.38, 95% CI 1.06–1.80). Similarly, those who had negative views toward control measures (e.g. mandatory quarantine) fared worse regarding their mental health. Unsurprisingly, those who reported experiencing greater difficulty with quarantine experienced substantially greater odds of developing poor mental health. For those who reported having a difficult or very difficult time with quarantine relative to participants who had no difficulty, their respective odds ratios were 13.7 (95% CI 11.3−16.8) and 27.9 (95% CI 22.2−35.4) of developing poor mental health. Those who believed that the quarantine was necessary had 64% reduced odds of developing poor mental health compared with those who did not (odds ratio 0.37, 95% CI 0.34−0.41). Those who believed that the 14-day quarantine was too long had almost three times greater odds of developing poor mental health (odds ratio 2.79, 95% CI 2.49−3.12).

**Table 4:** Multivariable adjusted odds ratios quantifying the relationship between developing poor mental health and COVID-19 beliefs and prevention behaviours

	Unadjusted			Adjusted^a^		
	Odds ratio	95% CI	*P*-value	Odds ratio	95% CI	*P*-value
Quarantine difficulty^b^						
Not difficult at all	–	–		–	–	
A little difficult	3.86	3.21–4.67	<0.001	3.71	3.08–4.49	<0.001
Difficult	14.5	11.9–17.7	<0.001	13.7	11.3–16.8	<0.001
Very difficult	29.6	23.6–37.4	<0.001	27.9	22.2–35.4	<0.001
I am not sure	4.37	3.25–5.87	<0.001	4.21	3.12–5.67	<0.001
Quarantine necessary (true)^c^	0.37	0.34–0.40	<0.001	0.37	0.34–0.41	<0.001
Quarantine is too long (true)^d^	2.73	2.46–3.03	<0.001	2.71	2.43–3.01	<0.001
Anxious about COVID-19^e^						
Never	–	–		–	–	
Sometimes	0.98	0.85–1.12	0.7	0.96	0.83–1.10	0.5
Often	1.25	1.05–1.48	0.010	1.18	0.99–1.41	0.057
All of the time	1.47	1.13–1.92	0.004	1.38	1.06–1.80	0.018
Mask-wearing^f^						
Always	–	–		–	–	
Usually	1.21	1.09–1.35	<0.001	1.17	1.05–1.31	0.004
Rarely	2.90	2.01–4.23	<0.001	2.98	2.06–4.36	<0.001
Avoid restaurants^g^						
Yes	–	–		–	–	
Usually	1.17	1.06–1.30	0.002	1.08	0.97–1.20	0.2
No	1.73	1.50–1.99	<0.001	1.58	1.37–1.82	<0.001
Hand-washing^h^	0.64	0.54–0.77	<0.001	0.68	0.56–0.81	<0.001
Seeing friends and family^i^	1.00	0.85–1.19	>0.9	0.95	0.80–1.13	0.6

a. All adjusted models control for age, gender and continent of origin.

b. Modelling the relationship between quarantine difficulty and developing poor well-being. Question: ‘How difficult are you finding the quarantine experience?’. The value taken was the last response between day 7 and day 14 questionnaires.

c. Modelling the relationship between the belief that quarantine is necessary and developing poor well-being. Question: ‘I think the 14-day quarantine is necessary and has worked’. Answer: true or false. The value taken was the last response between day 7 and day 14 questionnaires.

d. Modelling the relationship between the belief that quarantine is too long and developing poor well-being. Question: ‘I think the 14-day quarantine is too long’. Answer: true or false. The value taken was the last response between day 7 and day 14 questionnaires.

e. Modelling the relationship between feeling anxious about COVID-19 and developing poor well-being. Question: ‘I feel anxious or worried about COVID-19’. The value taken was the first response between day 7 and day 14 questionnaires.

f. Modelling the relationship between mask-wearing behaviour and developing poor well-being. Question: ‘I wear a mask when around other people’.

g. Modelling the relationship between avoiding restaurants and developing poor well-being. Question: ‘I avoid restaurants and bars now’.

h. Modelling the relationship between hand-washing and developing poor well-being. Question: ‘I wash my hands more often than I did before COVID-19’.

i. Modelling the relationship between seeing friends and family and developing poor well-being. Question: ‘I see my friends and family about as often as I did before COVID-19’.

#### Prevention behaviours

Similar patterns exist between the relationship of newly developed poor well-being and prevention behaviours. Those who seem to engage in fewer prevention behaviours exhibit a higher likelihood of poor mental health. For instance, travellers who reported rarely wearing masks or face coverings had nearly three times higher odds of developing poor mental health (odds ratio 2.98, 95% CI 2.06−4.36) (Table 4).

## Discussion

In Canada, as in many countries around the world, the government and public health officials imposed 14-day quarantines on those arriving at airports from foreign destinations, in an effort to stop the importation of COVID-19. Yet data suggest that quarantine is an imperfect approach as a public health measure, and its widespread and long-standing use in the current pandemic amounts to a global experiment. Previous research has demonstrated that quarantines are difficult to enforce, have variable compliance and may result in significant negative effects related to social isolation, restricted physical activity, lost productivity and income.^9,19^ Further, although research has demonstrated associations between quarantine and mental health distress,^8,9,26^ less is known about the change in mental health during quarantine,^16,17^ particularly when quarantine is imposed on specific individuals rather than the entire population.

This study sought to determine the impact of the quarantine period on the mental health well-being of travellers arriving at a major urban airport, and evaluate factors associated with greater declines in well-being among those suffering from the effects of quarantine. Results from self-report data provided by almost 11 000 people revealed that mental health well-being declined significantly between the onset of quarantine and day 7 of their quarantine. Consistent with studies of other populations facing lockdown and enforced social isolation, individuals who were most likely to develop poor mental health were younger and female.^4,11,26^ Those who developed poor mental health were also more likely to have arrived from Africa or Europe, whereas those arriving from Asia had significantly lower odds of developing poor mental health.

Attitudes about COVID-19 and measures imposed to control its spread also predicted the decline in mental health well-being. Specifically, those who felt that quarantine was necessary and accepted that 14 days was an appropriate length for quarantine were less likely to develop poor mental health, a finding that confirms that of cross-sectional design studies.^10,11,18^ Further, those who engaged in public health protection measures, which similarly suggests confidence in public health officials and the advice they provide, were also less likely to develop poor mental health well-being. It should be noted that during that period of time, scientific evidence regarding the spread of COVID-19 as reported in the print as well as social media, and daily press briefings by public health officials representing multiple levels of government in Canada, was highly contradictory. This could easily have fuelled scepticism and confusion among individuals subjected to quarantine, particularly younger individuals who were more closely tied to social media.^5^ Given previous research findings of increased mental health distress as a result of quarantine, and that trust in public officials affects quarantine compliance,^1,19^ this is an issue of serious concern.

### Limitations

This study was conducted at a single terminal at Toronto's Pearson International Airport, albeit representing the majority of international flights arriving at Canada's busiest airport. We enrolled approximately 20% of passengers. We believe that up to half of the arriving passengers would have either been exempt from quarantine or met our exclusion criteria, so our participation rate likely approaches 40%. We had losses to follow-up, and these may have biased the results if those who broke quarantine or dropped out of the study had higher or lower rates of distress. We also did not determine the purposes of participants’ travel, and are unable to assess whether factors such as ending vacation or attending the funeral of a family member may have affected mental health. Further, we do not know the nature of quarantine accommodations and so cannot assess whether experiences of confinement, such as hotel quarantine, results in poorer outcomes.

Well-being was measured with a self-report scale and was based on a limited number of items, thus selection bias owing to unmeasured factors may not be accounted for. The WHO-5 has been widely used for screening for psychological well-being in large populations and is not a diagnostic tool. However, researchers employing both the tool and clinical diagnoses have found that a score of <50% is consistent with a clinical diagnosis of depression.^23,24^ Finally, analyses that quantify relationships between attitudes and behaviours with declines in mental health are cross-sectional, and therefore reverse causation cannot be ruled out.

In conclusion, although the widespread use of quarantine may be effective in limiting the spread of COVID-19, the results of this study suggest that such measures may have a significant effect on mental health. The characteristics of those most likely to have the most significant declines in well-being suggest that public health officials need to more clearly communicate why public health measures are being implemented, to ensure individuals have confidence in the need for them. Individuals who believe in the value of such measures are less likely to have declines in mental health status during quarantine. Administration of brief screening tools to identify those at greatest risk of decline in mental health status and offer them appropriate supportive interventions are recommended. Psychiatry has a role to play in contributing to the public policy debate to ensure that all aspects of health and well-being are reflected in decisions to isolate people from others.

## Data Availability

Deidentified participant data will be made available with publication through an open-data repository at https://doi.org/10.5683/SP2/LOQ2HQ.
